# Metagenomics Versus Metatranscriptomics of the Murine Gut Microbiome for Assessing Microbial Metabolism During Inflammation

**DOI:** 10.3389/fmicb.2022.829378

**Published:** 2022-02-03

**Authors:** Juan Jovel, Aissata Nimaga, Tracy Jordan, Sandra O’Keefe, Jordan Patterson, Aducio Thiesen, Naomi Hotte, Michael Bording-Jorgensen, Sudip Subedi, Jessica Hamilton, Eric J. Carpenter, Béatrice Lauga, Shokrollah Elahi, Karen L. Madsen, Gane Ka-Shu Wong, Andrew L. Mason

**Affiliations:** ^1^Department of Medicine, University of Alberta, Edmonton, AB, Canada; ^2^Office of Research, Faculty of Medicine and Dentistry, University of Alberta, Edmonton, AB, Canada; ^3^Universite de Pau et des Pays de l’Adour, E2S UPPA, CNRS, IPREM, Pau, France; ^4^Department of Laboratory Medicine and Pathology, University of Alberta, Edmonton, AB, Canada; ^5^Department of Physiology, University of Alberta, Edmonton, AB, Canada; ^6^Department of Biological Sciences, University of Alberta, Edmonton, AB, Canada; ^7^School of Dentistry, University of Alberta, Edmonton, AB, Canada; ^8^BGI-Shenzhen, Beishan Industrial Zone, Shenzhen, China

**Keywords:** microbiome, gut inflammation, shotgun metagenomics, metatranscriptomics, bacterial metabolic pathways

## Abstract

Shotgun metagenomics studies have improved our understanding of microbial population dynamics and have revealed significant contributions of microbes to gut homeostasis. They also allow *in silico* inference of the metagenome. While they link the microbiome with metabolic abnormalities associated with disease phenotypes, they do not capture microbial gene expression patterns that occur in response to the multitude of stimuli that constantly ambush the gut environment. Metatranscriptomics closes that gap, but its implementation is more expensive and tedious. We assessed the metabolic perturbations associated with gut inflammation using shotgun metagenomics and metatranscriptomics. Shotgun metagenomics detected changes in abundance of bacterial taxa known to be SCFA producers, which favors gut homeostasis. Bacteria in the phylum Firmicutes were found at decreased abundance, while those in phyla Bacteroidetes and Proteobacteria were found at increased abundance. Surprisingly, inferring the coding capacity of the microbiome from shotgun metagenomics data did not result in any statistically significant difference, suggesting functional redundancy in the microbiome or poor resolution of shotgun metagenomics data to profile bacterial pathways, especially when sequencing is not very deep. Obviously, the ability of metatranscriptomics libraries to detect transcripts expressed at basal (or simply low) levels is also dependent on sequencing depth. Nevertheless, metatranscriptomics informed about contrasting roles of bacteria during inflammation. Functions involved in nutrient transport, immune suppression and regulation of tissue damage were dramatically upregulated, perhaps contributed by homeostasis-promoting bacteria. Functions ostensibly increasing bacteria pathogenesis were also found upregulated, perhaps as a consequence of increased abundance of Proteobacteria. Bacterial protein synthesis appeared downregulated. In summary, shotgun metagenomics was useful to profile bacterial population composition and taxa relative abundance, but did not inform about differential gene content associated with inflammation. Metatranscriptomics was more robust for capturing bacterial metabolism in real time. Although both approaches are complementary, it is often not possible to apply them in parallel. We hope our data will help researchers to decide which approach is more appropriate for the study of different aspects of the microbiome.

## Introduction

The human microbiome, which includes bacteria, archaea, eukaryotic viruses, virophages, and fungi, is tightly linked to host health. Quantitative and qualitative fluctuations in the composition of microbial communities correlate with disease. Hence, the microbiome is a potential source of novel therapeutics (Jovel et al., 2018a). Notwithstanding its importance, until recently, little was known about the microbiome, including taxonomy, assemblage into communities and metabolic contributions to host physiology (Davenport et al., 2017). Microbiome studies have been boosted by high-throughput parallel sequencing technologies and associated bioinformatics approaches. Metagenomic studies aim at cataloging genes and other sequences that are contained in microorganisms’ genomes and therefore have the potential to infer the microbiome’s functional capability (Nayfach et al., 2015); however, they may be inadequate to portray the spatio-temporal patterns of gene expression that occur in response to environmental stimuli like xenobiotics, dietary changes, or pathogens invasion. Studying the taxonomic composition of microbial communities provides insights into their diversity and richness, while recent metatranscriptomics and metabolomic studies have revealed that a functional redundancy is present among related bacterial taxa and that such redundancy is an important component of host’s fitness since function can be preserved despite perturbations that alter bacterial populations’ structure (Sharpton, 2018). Therefore, pure metagenomics surveys associated with phenotypes have the intrinsic risk of erroneously associating microorganisms with protective or detrimental roles in diseases. The ultimate goal is to make causal inferences regarding the observed phenotype based on measurements of microbes’ activity and/or population dynamics.

To evaluate the suitability of shotgun metagenomics as well as metatranscriptomics for assessing changes in metabolic pathways of the gut microbiome, we used a mouse dextran sodium sulfate (DSS) acute inflammation model. Mouse models have been instrumental for the study of the gut microbiome and have provided valuable insights into host-microbiome interactions, many of which may be extensible to humans (Hugenholtz and de Vos, 2018). Shotgun metagenomics sequences are usually compared against reference databases through alignments to clade-specific marker genes (Segata et al., 2012), pseudo-alignments to full or partial genomes (Wood and Salzberg, 2014) and *bona fide* alignments to full or partial genomes (Huson et al., 2007). It is also possible to make functional inferences from shotgun libraries using approaches like HUMAnN, which align *in silico* translated metagenomics sequences to a protein database and then assign genes to pathways using MinPath and finally calculate pathways’ coverage (Abubucker et al., 2012; Franzosa et al., 2018). Metatranscriptomics libraries are derived directly from cDNAs of mRNA transcripts and hence represent a snapshot of gene expression. They are aligned to protein databases using sensitive but computationally expensive alignments (Abubucker et al., 2012; Nayfach et al., 2015) or more sophisticated methods, like those incorporating hidden Markov models (Remmert et al., 2011). In both cases, the extent and accuracy of sequence classification is dependent on completeness and quality of the reference database; this implies that significant improvements in sequence classification should be expected as databases exponentially increase in size, and as bioinformatics tools are improved, which also allows automated curation of databases.

Gut inflammation is a relatively well-characterized phenotype, where many functional alterations of the microbiome are known. One of the main contributions of the gut microbiome to gut physiology is the anaerobic fermentation that results in the production of short-chain fatty acids (SCFA) including acetate (C_2_), propionate (C_3_), and butyrate (C_4_). They are involved in many essential processes like energy supply to intestinal epithelial cells (Vinolo et al., 2011), signaling through activation of GPCR receptors (Brown et al., 2003), regulation of many cytokines (TNFα-, IL-2, IL-6, and IL-10), and the migration of leukocytes to sites of inflammation to destroy invading pathogens (Schirmer et al., 2016). The microbiome is also a source of immune modulators including elicitors of Toll-like receptors such as lipopolysaccharides and flagellin (Vijay-Kumar et al., 2010) and ATP, which induces the differentiation of CD4 + Th17 immune cells (Atarashi et al., 2008). Thus, alteration in the proportions of microorganisms and their gene products in the gut affects the patterns of production and release of microbial metabolites in the lumen often resulting in disease (Vernia et al., 1988; Huda-Faujan et al., 2010), hence the importance of assessing microbiome functional activity.

Although inflammation is a well-known phenotype and so are the methods used in this paper, our interest was specifically in comparing the ability of shotgun metagenomics and metatranscriptomics to assess functional changes in the microbiome. We predicted proteins encoded by the metagenome detected in shotgun libraries, and directly measured mRNA abundance in the gut microbiome by metatranscriptomics. Although each of those approaches have the ability to detect bacterial proteins, we hypothesized that shotgun metagenomics does not have the sensitivity to detect statistically significant changes in protein abundance occurring in response to inflammation. Our hypothesis was proven to be correct. The goal of this study was to illustrate, using the mouse dextran sodium sulfate acute inflammation model, the advantages and limitations of each of the approaches to make rational inferences about the phenotype under study, all in the spirit of assisting metagenomics basic and clinical researchers during their decision-making process.

## Results

### Assessment of Gut Inflammation

**FIGURE 1:**
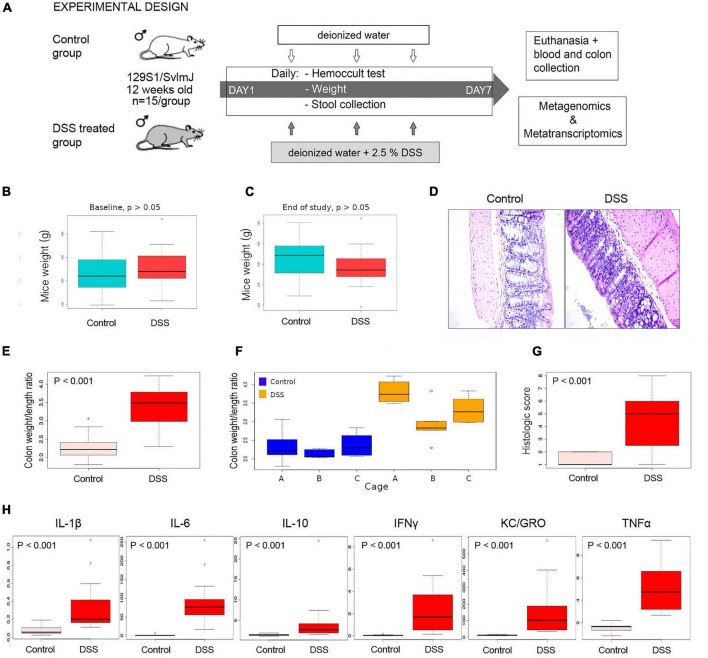
Inflammation experimental design and assessment of inflammation. **(A)** 129S1/SvlmJ mice (Jackson Laboratories) were acclimatized in our mice facility for 2 weeks and at 12 weeks of age were divided into two halves (*n* = 15 each). All animals were fed with a conventional Shaw diet for the whole period of the experiment. One-half was subjected to a solution of 2.5% of DSS in drinking water, while control animals received only water. Stools were collected daily, and a Hemoccult test was conducted until day 7, when animals were euthanized, and blood and colon tissue were collected. Metagenomics and metatranscriptomics libraries were constructed from stools collected at day 0 and day 7 and sequenced using Illumina technology. **(B,C)** Mice weight did not significantly change during the course of the study, suggesting that the physiological stress of animals was not extreme. **(D)** Colon tissue stained with H&E and photographed under light microscopy at 40X. **(E)** Statistical comparison of the colon weight/length ratio in control and DSS-treated animals at end of study (day 7). **(F)** Variability between cages. **(G)** Statistical comparison of a histologic score in control and DSS-treated animals at end of study (day 7). **(H)** Statistical comparison of abundance of a series of pro-inflammatory cytokines, determined in a Meso Scale Discovery assay, in control and DSS-treated animals at end of study (day 7).

We used a dextran sodium sulfate (DSS) mice inflammation model (Figure 1A) to profile metabolic pathways by shotgun metagenomics and metatranscriptomics. None of the DSS-treated animals significantly lost weight during the course of our experiment (Figures 1B,C). Histological analyses demonstrated that inflammation had been effectively induced in the colon of animals treated with DSS (Figure 1D); for instance, the structure of the villi was distorted and higher infiltration of inflammatory cells into the villi was observed in DSS-treated animals. The colon weight/length ratio was significantly higher in DSS-treated than in control animals (Figure 1E). However, a considerable cage effect was observed, suggesting that inflammation in animals of cage A was more severe than in the other cages (Figure 1F). A pathology histologic score indicative of inflammation was also significantly elevated in DSS-treated animals (Figure 1G). Screening of six pro-inflammatory cytokines also showed that inflammation took place in DSS-treated animals (Figure 1H).

**FIGURE 2:**
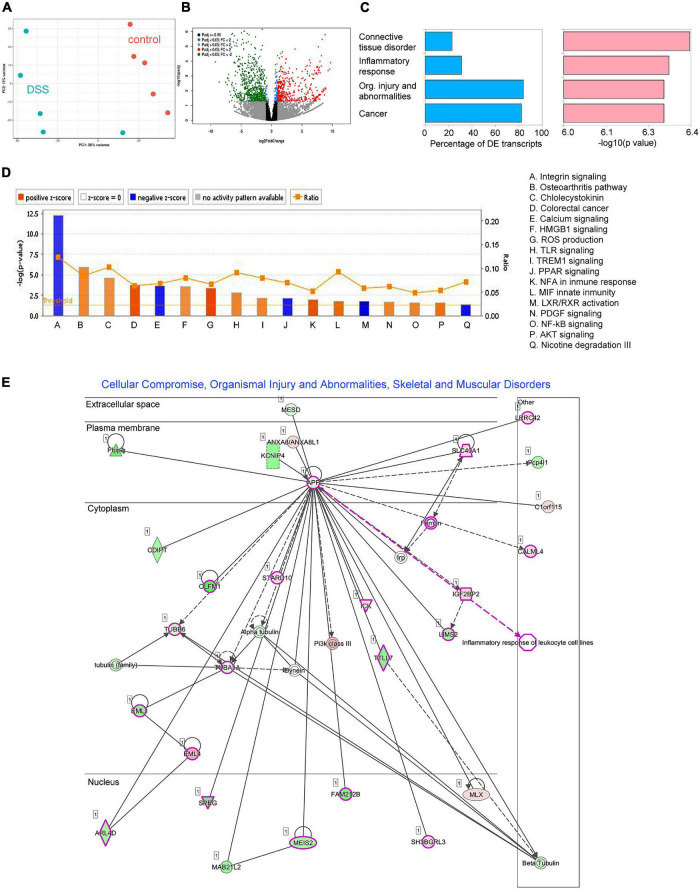
Differential expression analysis results for RNAseq data comparing distal colon of five control and five DSS-treated animals. **(A)** Principal component analysis (PCA) on Euclidean distances calculated on counts data subjected to a regularized logarithmic transformation. **(B)** Volcano plot showing deregulated transcripts (*p* < 0.01; fold-change > 2). Red dots are significantly upregulated transcripts, while green dots are significantly downregulated transcripts. **(C)** Diseases identified by ingenuity pathways analysis (IPA). Blue bars represent the percentage of differentially expressed transcripts related to a disease, while pink bars represent the significance (expressed in –log10 of the *p*-value). **(D)** Canonical IPA pathways deregulated in inflamed tissue. **(E)** The most deregulated metabolic network identified by IPA based on deregulated transcripts: *Cellular compromise, organismal injury, and abnormalities*. Genes shown in green were found downregulated while genes shown in red were found up regulated. Genes involved in gastrointestinal disease are highlighted in pink.

On day 7, we also conducted RNA-Seq on distal colon tissue of five animals treated with DSS and five controls (Figure 2). Description of all libraries generated in this study is presented in Supplementary Table 1. In total, we found expression changes in 537 transcripts (198 upregulated and 339 downregulated) (Figures 2A,B and Supplementary Table 2), many of those transcripts have been found implicated in connective tissue disorder, inflammatory response, cancer and organismal injury and abnormalities (Figure 2C), among others (Figure 2D). The most deregulated disease network was “Cellular compromise organismal injury and abnormalities,” which also includes genes involved in gastrointestinal disease (Figure 2E). All diseases and functions deregulated are shown in Supplementary Table 3. Some typical pro-inflammatory genes were found upregulated including KC-GRO (CXCL1), TNFα, IL-1β, and IL-1α (58-, 31-, 436-, and 121-fold, respectively; see Supplementary Table 2).

### Shotgun Metagenomics Revealed Perturbation of the Microbiome but Not of Microbial Functions

**FIGURE 3:**
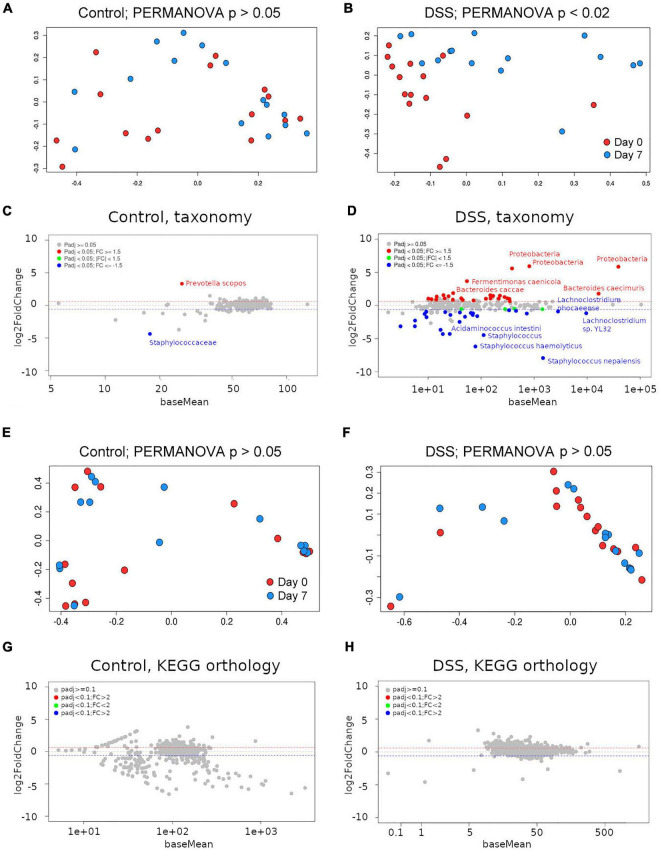
Assessment of the gut metagenome by shotgun metagenomics. **(A)** Principal coordinates analysis (PCoA) using Bray-Curtis distances on Kraken2-derived taxa abundance from control or **(B)** DSS-treated animals between day 0 and day 7. PERMANOVA *p*-values are included. MA plots depicting taxonomic differential accumulation analysis results for control **(C)** or DSS-treated **(D)** animals. Principal coordinates analysis (PCoA) using Bray-Curtis distances on HUMAnN2 KEGG orthology groups abundance from control **(E)** or DSS-treated **(F)** animals between day 0 and day 7. PERMANOVA *p*-values are included. MA plots depicting KEGG orthology differential abundance analysis results for control **(G)** or DSS-treated **(H)** animals.

Principal coordinate analysis (PCoA) on Bray-Curtis distances of taxa abundance per sample showed that the microbiome of DSS-treated, but not of control, animals had changed from day 0 to day 7 (PERMANOVA *p*-value < 0.02; Figures 3A,B). All bacterial taxa detected with abundance equal or greater to 10 reads per sample (on average) are described in Supplementary Table 4. To statistically analyze differences in bacterial taxa abundance between groups (control vs. DSS-treated), we modeled observed abundance using a negative binomial distribution after scaling the data to account for sampling depth (Lin and Peddada, 2020). In differential accumulation analysis, with DESeq2, comparing the baseline and end-of-study time points and using corrected *p*-values < 0.05 as a significance threshold, only two taxa were found at different abundance (corrected *p* < 0.05). Namely, abundance of *Prevotella scopos* and the family *Staphylococcaceae* were found upregulated and downregulated, respectively (Figure 3C and Supplementary Table 5). We therefore assumed that, somehow, changes over time in those bacteria occur due to stimuli other than inflammation. In DSS-treated animals, more dramatic changes were observed. Twenty-seven taxa increased their abundance including bacteria in the *Halomonadaceae* and *Alcanivoracaceae* families, as well as species like *Bacteriodes caccae* and *Prevotella denticola* (Figure 3D and Supplementary Table 6). Thirty taxa decreased their abundance over the course of the experiment (Figure 3D and Supplementary Table 6). This included many bacteria in the families *Lachnospiraceae*, *Clostridiaceae*, and *Peptococcaceae* (all in the Firmicutes phylum). In summary, a predominant decrease in Firmicutes and a predominant increase of Proteobacteria and Bacteroidetes was observed in DSS-treated animals.

Shotgun metagenomic sequences were subjected to *in silico* bacterial pathways profiling using the software HUMAnN2 (Abubucker et al., 2012). Surprisingly, PCoA on Bray-Curtis distances among KEGG orthology groups’ abundance per sample did not show significant differences between day 0 and day 7 neither in control nor in DSS-treated animals (Figures 3E,F; PERMANOVA *p*-value > 0.05). In differential abundance analysis, neither the control nor the DSS-treated animals showed any differentially accumulated KEGG orthology group (Figures 3G,H). Thus, despite considerable differences in taxa abundance, no differences could be detected in KEGG orthology groups predicted from each (control or DSS-treated) metagenome.

Because inflammation was more severe in cage A, we repeated this analysis for only animals in such a cage and obtained similar results (data not shown). Thus, not even for the most severely inflamed animals we detected differences in predicted gene content. We also decreased the level of astringency, considering adjusted *p*-values < 0.1 to be significant, but no KO group showed statistically significant differences between control and DSS-treated mice.

### Metatranscriptomics Revealed Dysregulation of Gene Families Related to Gut Homeostasis

**FIGURE 4:**
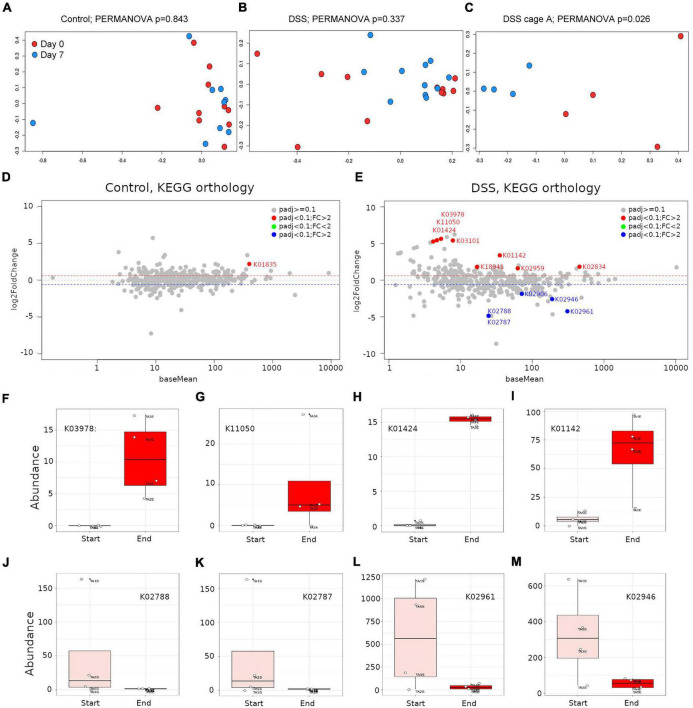
Assessment of the gut microbiome transcripts abundance by metatranscriptomics. Principal coordinates analysis (PCoA) using Bray-Curtis distances on HUMAnN2-derived gene families abundance derived from control or DSS-treated animals between day 0 and day 7. PERMANOVA *p*-values are included; **(A)** control animals including all cages, **(B)** DSS-treated animals including all cages, **(C)** DSS-treated animals for only cage A, where inflammation was strongly recorded. MA plots depicting KEGG orthology differential abundance analysis results for control **(D)** or DSS-treated **(E)** animals. **(F–M)** Representative boxplots of KEGG orthology groups differentially accumulated in DSS-treated animals, at the beginning (rose boxes) or end (red boxes) of the study. KEGG orthology groups abundances were subjected to a regularized logarithmic transformation before boxplots construction.

In PCoA and PERMANOVA analyses, neither control (Figure 4A) nor DSS-treated (Figure 4B) animals showed a significant difference in accumulation of KEGG orthology groups between baseline and end-of-study timepoints (*p* > 0.05). In the DSS-treated samples PCoA plot, some points deviated from the general pattern. A closer inspection revealed that such samples belonged to cage A, in which inflammation was apparently induced more severely (Figure 1F). PCoA and PERMANOVA analyses showed that, for cage A alone, significant differences in the gene expression profile of DSS-treated animals were detectable when comparing baseline and end-of-study timepoints (Figure 4C; PERMANOVA *p* < 0.05). Analogous comparisons in other cages did not reveal any statistically significant difference (not shown).

HUMAnN2 results were subjected to differential abundance analysis for only samples in cage A as above, but using as significance threshold of 0.1 (corrected *p*-values < 0.1). In control animals, the only KEGG orthology group upregulated was KO1835, which corresponds to a phosphoglucomutase (Figure 4D and Supplementary Table 7). No KEGG orthology group was found downregulated in control animals (Figure 4D). Conversely, in DSS-treated animals, eight KEGG orthology groups were found dramatically upregulated (fold-change ranging from 3 to 50) and five KEGG orthology groups were found downregulated (Figure 4E and Supplementary Table 8). The five most upregulated KEGG orthology groups in the gut of DSS-treated animals were K03978 (GTP-binding protein; fold change = 50); K11050 (Multidrug/hemolysin transport system ATP-binding protein; fold change = 43), K01424 (L-asparaginase; fold change = 42); K03101, which represents a bacterial signal peptidase II (fold change = 39) and K01142 (exodeoxyribonuclease III; fold change = 10). The five downregulated KEGG orthology groups included two PTS system, lactose-specific components (IIC and IIB; K02788 and K02787; fold change = 29), two small subunit ribosomal proteins (S17 and S10; K02961 and K02946; fold change = 19 and 6, respectively) and the large subunit ribosomal protein L3 (K02906; fold change = 4). In Figures 4F–M the abundance of selected proteins (KO groups) are depicted. Here, it is noticeable that upregulated proteins showed a more consistent pattern among animals in each group, and the same was more variable for downregulated proteins. In summary, metatranscriptomics libraries proved to be more informative than metagenomics ones at profiling bacterial metabolic pathways, as discussed below.

### Costs of Implementation

In Table 1, we present a summary of costs associated with the production and sequencing of each type of libraries discussed in this article. Monetary costs and labor time were substantially lower for shotgun metagenomics than for metatranscriptomics libraries. Costs for bioinformatics are not included but are similar for both types of libraries, since they were analyzed with similar pipelines, and are dependent on sequencing depth.

**TABLE 1:** Comparative analysis of costs for implementation of shotgun metagenomics and metatranscriptomics.

Description	Cost (US dollars)
**Shotgun metagenomics**	
DNA extraction (FastDNA Fungal/Bacterial DNA kit, MP Biomedicals; 96 Rx)	$ 10.54
Consumables (tubes, tips, gloves, etc.)	$ 5.00
Nextera XT reagents (96 Rx)	$ 45.61
Illumina indexing oligos	$ 3.00
Agencourt AMPure XP beads (Beckman Coulter)	$ 2.00
QC reagents (Bioanalyzer + Qubit)	$ 8.00
Sequencing at 1 M reads (150 bp paired-end)	$ 15.00
Hands-on time	0.3 h
**Total cost per sample**	**$ 86.15**
**Metatranscriptomics**	
RNA extraction (FastRNA spin kit, MP Biomedicals; 96 Rx)	$ 12.60
Consumables (tubes, tips, gloves, etc.)	$ 5.00
ScriptSeq Complete Kit (Epicenter; 48 Rx)	$ 179.47
Agencourt AMPure XP beads (Beckman Coulter)	$ 2.00
QC reagents (Bioanalyzer + Qubit)	$ 8.00
Sequencing at 3 M reads (150 bp paired-end)	$ 30.00
Hands-on time	1.5 h
**Total cost per sample**	**$ 237.07**

*Costs include construction of libraries and sequencing, but not bioinformatics.*

## Discussion

A balanced microbiota is associated with gut homeostasis; therefore, detecting imbalances of the microbiome at the taxonomical and/or functional levels provides insights into disease phenotypes. Taxonomic profiling through metagenomics is informative insofar as bibliographic information exists to correlate fluctuations in taxa abundance with reported functional roles of such taxa. Metagenomics data also allows computational inference of the coding capacity of the microbiome thus enabling statistical comparisons of the metagenome between classes under study. However, the whole bacterial gene complement is not constitutively expressed, instead, genes are turned on and off in response to a complex array of gut environmental clues. Metatranscriptomics can detect those selective patterns of gene expression but its implementation is more expensive and troublesome.

A major dilemma of the gut immune system is to be ready to react against pathogens whilst being tolerogenic to the presence of a multitude of commensal microorganisms’ and food antigens. Rapidly accumulating evidence supports the notion that the gut microbiota is intimately linked to intestinal inflammation or to the absence thereof. Consequently, colitis does not fully develop in germ-free mice (Macdonald and Monteleone, 2005; Gkouskou et al., 2014). One possibility is that CD4 + T cells reactive to enteric bacterial antigens mediate inflammatory processes (Macdonald and Monteleone, 2005). Although no conclusive evidence exists yet, some pro-inflammatory and anti-inflammatory roles have been attributed to specific bacteria (Lee and Kim, 2017). For instance, a marked decrease in abundance in bacteria of the phylum Firmicutes and an increase in *Enterobacteriaceae* have been reported in inflammatory bowel disease, and transcriptional activity mirrors such relative abundance (Rehman et al., 2010; Morgan et al., 2012). Butyrate-producing bacteria like *Roseburia hominis* and *Faecalibacterium prausnitzii* are thought to have a protective role against inflammation and their abundance is reduced in ulcerative colitis patients (Machiels et al., 2014; Abu-Ali et al., 2018; Schirmer et al., 2018). In general, decreased carbohydrate metabolism and amino acids biosynthesis with concurrent increase in nutrient transport and uptake have been observed during inflammation (Morgan et al., 2012; Franzosa et al., 2014).

We analyzed the feasibility of using shotgun metagenomics as a surrogate of metatranscriptomics to assess the metabolic changes of the microbiome during induction and/or maintenance of disease phenotypes (i.e., inflammation). Metagenomics is cheaper than metatranscriptomics and is often used as a proxy to estimate bacterial protein abundance in the gut. In the shotgun metagenomics survey of stools from DSS-treated animals, significant changes in bacteria relative abundance, from days 0 to 7, were observed (Supplementary Table 6). The most significant changes revealed a marked decrease of Firmicutes and a concomitant increase in Bacteroidetes and Proteobacteria. Considering findings reported in the literature, the bacterial taxa with decreased abundance in this group are likely related with colonocyte homeostasis, and therefore their depletion will favor progression of inflammation in the gut. For instance, a series of Clostridia were found at reduced relative abundance, which have been implicated in gut homeostasis (Lopetuso et al., 2013). More specific findings included butyrate-producing bacteria like the species *Intestinomonas butyriciproducens* (Kläring et al., 2013), the acetate-converting butyrate producers *Eubacterium rectale* (Rivière et al., 2015), *Christensenella massiliensis*, and a series of bacteria in the family *Lachnospiraceae*. Bacteria in the family *Christensenellaceae* have been associated with gut health, and their reduction has been reported associated with negative effects in obesity and inflammatory bowel disease (Waters and Ley, 2019), while bacteria in the family *Lachnospiraceae* are among the main producers of SCFA (Deleu et al., 2021). Thus, interpreting bacterial abundance changes in light of published reports allows inference of the role of specific bacterial taxa during inflammation. Interestingly, when the shotgun libraries were used to infer the metagenome, with HUMAnN2, differences in the KEGG orthology groups’ abundance were not statistically significant (Figures 3G,H). These results agree with a reported functional redundancy in the metagenome of the gut microbiome (Franzosa et al., 2014). How can it be that evident changes in taxonomy profiles did not result in differences in abundance of predicted functions? One possibility is that, as significant changes in specific taxa occur, this is compensated by opposite changes in other taxa with redundant genetic complements. Also, as shotgun metagenomics libraries include reads along the whole bacterial genomes, the proportion of reads per library mapping to protein coding sequences is smaller than in metatranscriptomics libraries for equal amounts of sequencing data; this should lower the sensitivity of shotgun metagenomics libraries when assessing gene’s abundance through comparison against reference sequences. We also explored possible changes in microbial diversity between the baseline and the end of the experiment, but neither the control nor the DSS-treated animals experienced changes in microbial diversity during the course of the experiment (Wilcoxon test between Simpson or Shannon diversity indices at baseline and end of study were not significant; *p* > 0.05; data not shown). In principle, the apparently lower sensitivity of metagenomics libraries to detect statistically significant changes in KEGG orthology groups might be attributed to reduced sequencing. However, the number of KEGG orthology groups that were surveyed in metagenomics libraries at a depth of 10 or more reads per sample (on average) was slightly higher than the ones in the metatranscriptomics libraries (data not shown); this suggests that the underlying problem is not derived from scarce alignments of metagenomics sequences to protein reference sequences.

In metatranscriptomics, a direct readout of gene activity is collected. Only minor differences were observed at the metatranscriptomics level when all animals in each group were considered. However, as mentioned above, a strong cage-effect was detected, animals in cage A were more severely inflamed. Comparisons between day 0 and day 7 time points for cage A alone showed statistically significant differences (padj < 0.1) in DSS-treated animals. Here, thirteen KEGG orthology groups were found at altered abundance and such changes might represent complex interactions between the microbiome and the inflamed gut. The most upregulated KEGG orthology group in the gut of DSS-treated animals was a GTP-binding protein. It is known that GTP-binding proteins are activated during inflammation and they amplify immune responses and regulate tissue damage (Tretina et al., 2019), this may hint to the activity of some protective bacteria. Multidrug/hemolysis transport system ATP-binding protein was also found upregulated and it may be implicated in bacterial pathogenesis (Lewis et al., 2012), perhaps as a reflection of the increase in Proteobacteria. As mentioned above, inflammation is associated with an increase in nutrient transport (Morgan et al., 2012; Franzosa et al., 2014). Multidrug/hemolysis transport system ATP-binding protein and the signal peptidase II may contribute to nutrient transport. In addition, an L-asparaginase was also found at increased abundance. Such enzymes have long been used as anti-cancer agents (Vimal and Kumar, 2018) and their antiinflammatory role has also been addressed from the perspective of immune cells suppression (Song et al., 2017). Another enzymes upregulated, although to a lesser extent, included a exodeoxyribonuclease III (K01142), the ribosome binding factor A (K02834) and the two-component system, OmpR family response regulator ArlR (K18941), which, at least in *Staphylococcus aureus*, is involved in adhesion, biofilm formation, and virulence (Ouyang et al., 2019). The five downregulated KEGG orthology groups corresponded to three ribosomal proteins and two proteins involved in lactose transport (PTS system, lactose-specific components, IIB and IIC), which were downregulated to the same extent since they are co-expressed as part of the lac operon (Sanganeria and Bordoni, 2020).

In summary, shotgun metagenomics detected changes in bacterial taxa abundance that were informative in the context of inflammation, but failed to predict statistically significant changes in abundance of *in silico* translated proteins. The obvious explanation for such insensitivity is that shotgun metagenomics surveys gene content, rather than gene expression. Therefore, changes in microbial population composition will correlate with gene expression profiles only in the absence of functional redundancy in bacterial genomes. This is congruent with the fact that shotgun metagenomics was developed for surveying taxonomic composition and relative abundance of bacterial populations. Predicting the metagenome through *in silico* translation of metagenomics sequences is a bonus and can certainly be used as a proxy of gene abundance, but caution should be exercised when extrapolating results to gene expression profiles.

Although metagenomics and metatranscriptomics approaches are informatively complementary because together they provide snapshots of microbial taxonomy profiles, functional potential, and gene activity, it is not always feasible to interrogate the microbiome with the concurrent application of both approaches. There are situations where *the song is more important than the singer* and in such cases metatranscriptomics fits best. Conversely, in other scenarios, like those intended to identify microorganisms with therapeutic or pre- or probiotic potential, metagenomics is essential. Both approaches have their own intricacies: they vary in terms of costs, experimental complexity and analytical methods required. We hope that this comparative analysis assists researchers in selecting the most appropriate method to evaluate their hypotheses; as well as facilitate their implementation through the analytical pipelines included.

## Materials and Methods

### Animal Experiments

Mice were handled in accordance with the Basel Declaration and the International Council for Laboratory Animal Science (ICLAS) following a protocol approved by the Health Science Animal Care and Use Committee of the University of Alberta.

We generated original sequencing data from the microbiome of male mice stools, in which intestinal inflammation was induced by administering water containing 2.5% Dextran sodium sulfate (DSS) for 7 days (*n* = 15). Animals that received water without DSS served as controls (*n* = 15). Each group was distributed into three cages (A, B, and C; five animals per cage). From day 0, stools were collected daily and a hemoccult test was conducted. Stool collected on day 0 (baseline) and day 7 (end-of-study), were used to produce metagenomics and metatranscriptomics next generation sequencing (NGS) libraries. On day 7, animals were euthanized and dissected. Stool, blood, and colon samples were collected. All experiments were conducted in accordance with the ARRIVE guidelines.

### Metagenomics

Bacterial DNA was extracted with FastDNA™ Spin Kit for Microbes (MP-Biomedicals). We conducted shotgun metagenomics sequencing, using the NexteraXT technology (Illumina) for library preparation as described by the manufacturer. Libraries were sequenced using the NextSeq platform (Illumina), with a 300 cycles protocol. Libraries stats are presented in Supplementary Table 1.

Taxonomic classification of sequences was conducted with Kraken2 (Wood and Salzberg, 2014). The coding potential of the microbiome was then determined from shotgun sequencing using HUMAnN2 (Abubucker et al., 2012). Principal coordinate analysis and PERMANOVA were conducted in R. Differential abundance analysis was conducted with DESeq2 as described in Weiss et al. (2017) and Lin and Peddada (2020). For metagenomics comparisons, differences in taxa abundance were considered statistically significant if adjusted *p*-values < 0.05. In metatranscriptomics, because we only analyzed data for cage A, thereby the degrees of freedom are reduced, adjusted *p*-values < 0.1 were considered statistically significant.

### Metatranscriptomics

Bacterial RNA was extracted using FastRNA™ Spin Kit for Microbes (MP-Biomedicals). Essentially, preparation of metatranscriptomics libraries includes two steps: removal of the omnipresent ribosomal (r)RNA transcripts and ligation of previously fragmented reverse-transcribed messenger transcripts into sequencing adapters. We used the ScriptSeq™ Complete Kit (Bacteria) from Epicenter (Illumina). We recommend processing not more than six samples in parallel to be able to efficiently implement the protocol.

#### rRNA Depletion

For rRNA removal, paramagnetic beads conjugated to probes complementary to bacterial ribosomal RNA were washed with RNase-free water, resuspended in bead resuspension reagent and supplemented with 1 μl of RNase inhibitor. Two microgram of total bacterial RNA resuspended in 28 μl of RNase-free water were supplemented with 4 μl of RiboZero buffer and 8 μl of rRNA RiboZero removal solution, incubated 10 min at 68°C and finally equilibrated to room temperature (RT) for 5 min. Sixty-six microliter of pre-washed beads were added and mixed up by pipetting 10X, vortexed and incubated 5 min at RT. Samples were vortexed again and incubated 5 min at 50°C and immediately placed on a magnetic rack until the solution cleared up. Ninety microliter of the supernatant containing the rRNA-depleted RNA were removed, and such eluat was placed back on the magnetic rack for a second round of elution in 85 μl to remove residual beads. Elution of purified RNA was conducted in a RNeasy column (Qiagen). Volume of sample was adjusted to 100 μl with RNase-free water, supplemented with 350 μl of RLT buffer and 550 μl of absolute EtOH, and passed through the column in halves by centrifugation for 15 s at 8,000 × g. Column was supplemented with 500 μl of RPE buffer and centrifuged as before. Column was supplemented with 500 μl of 80% EtOH, centrifuged once at full speed for 2 min, transferred into a new tube and centrifuged again at full speed for 5 min. Column was transferred to a new tube and RNA was eluted in 12 μl of RNase-free water by centrifuging 1 min at full speed.

#### RNA Fragmentation and cDNA Synthesis

Nine microliter of purified RNA were supplemented with 1 μl of RNA fragmentation solution and 1 μl of cDNA synthesis primer and incubated at 80°C for 5 min and immediately chilled on ice. For first strand synthesis, the RNA-fragmentation reaction on ice was supplemented with a previously prepared mix containing 3 μl of cDNA synthesis premix, 0.5 μl of 100 mM DTT and 0.5 μl of StarScript RT and incubated 5 min, 25°C; 20 min, 42°C; 37°C (add 1 μl of finishing solution) and allow incubation for 10 min; 3 min, 95°C; hold at 25°C. For second strand synthesis, 7.5 μl of Terminal Tagging Premix was mixed with 0.5 μl of DNA polymerase and added to the RT reaction at 25°C and further incubated for 15 min. Second-strand synthesis reaction was inactivated at 95°C for 3 min and then transferred to ice. cDNA was purified with 45 μl of Agencourt AMPure XP beads (Beckman Coulter), incubating 15 min at RT, clearing up the solution for 5 min on a magnetic rack, removing the liquid phase and washing beads twice with 200 μl of 80% EtOH. Beads were air-dried on-rack, resuspended in 25 μl of RNAse-free water, solution cleared-up on-rack, and finally cDNA was recovered in 22.5 μl of the aqueous phase.

#### Indexing and Amplification

A mix containing 25 μl of FailSafe PCR mix E, 1 μl Forward PCR primer and 0.5 μl FailSafe PCR Enzyme, was added to the cDNA solution and 1 μl of a unique index added to each sample. The following indexing/amplifying reaction was conducted: 1 min, 95°C; 20X (30 s, 95°C; 30 s, 55°C; 3 min, 68°C); 7 min 68°C; hold at 4°C. PCR reaction was finally cleaned up with 50 μl of Agencourt AMPure XP beads as above. Libraries were sequenced on a HiSeq 2500 instrument (Illumina) following a 300-cycle paired-end protocol.

Metatranscriptome sequences were analyzed with HUMANn2 and statistical analysis conducted as described above.

### RNAseq in Colon Tissue

Total RNA from colon sections from control or inflamed animals was extracted with TRIzol reagent. RNAseq libraries were constructed using the TruSeq RNA Library Prep Kit v2 (Illumina), according to manufacturer’s instructions. Briefly, polyadenylated transcripts were enriched by pulling down RNA harboring sequences complementary to oligo-dT sequences, conjugated to paramagnetic beads. Long polyadenylated transcripts were chemically fragmented and cDNA was obtained by retrotranscription of fragmented transcripts. Resulting cDNA fragments were blunted and A-tailed and finally adapters were ligated which served as binding site of primers used for indexing PCR. Libraries were sequenced in a MiSeq instrument using a protocol with 75 cycles that included demultiplexing of samples according to their barcodes. Sequencing data was analyzed as previously described (Jovel et al., 2018b), and pathways analysis was conducted with the Ingenuity Pathways Analysis software (IPA; Qiagen).

## Data Availability Statement

The datasets presented in this study can be found in online repositories. The names of the repository/repositories and accession number(s) can be found in the article/Supplementary Material.

## Ethics Statement

The animal study was reviewed and approved by the Health Science Animal Care and Use Committee of the University of Alberta.

## Author Contributions

JJ, SO’K, JP, NH, and EC: study conceptualization. AN, TJ, SO’K, and NH: mice experiments. AN, TJ, SO’K, SS, and JH: library construction and sequencing. AN, TJ, SO’K, AT, NH, and MB-J: tissue histology and cytokine biochemistry. JJ, JP, and EC: analysis of data. JJ, BL, SE, KM, AM, and GW: interpretation of results and preparation of the manuscript. All authors read and approved the manuscript.

## Conflict of Interest

The authors declare that the research was conducted in the absence of any commercial or financial relationships that could be construed as a potential conflict of interest.

## Publisher’s Note

All claims expressed in this article are solely those of the authors and do not necessarily represent those of their affiliated organizations, or those of the publisher, the editors and the reviewers. Any product that may be evaluated in this article, or claim that may be made by its manufacturer, is not guaranteed or endorsed by the publisher.
